# Plant growth-promoting activity and quorum quenching-mediated biocontrol of bacterial phytopathogens by *Pseudomonas segetis* strain P6

**DOI:** 10.1038/s41598-020-61084-1

**Published:** 2020-03-05

**Authors:** Miguel Rodríguez, Marta Torres, Lydia Blanco, Victoria Béjar, Inmaculada Sampedro, Inmaculada Llamas

**Affiliations:** 1 Department of Microbiology, Faculty of Pharmacy, University of Granada, Granada, Spain; 2 Institute of Biotechnology, Biomedical Research Center (CIBM), University of Granada, Granada, Spain; 3 Institute for Integrative Biology of the Cell (I2BC), CEA/CNRS/University Paris-Sud, University Paris-Saclay, Gif-sur-Yvette, France

**Keywords:** Microbiology, Applied microbiology

## Abstract

Given the major threat of phytopathogenic bacteria to food production and ecosystem stability worldwide, novel alternatives to conventional chemicals-based agricultural practices are needed to combat these bacteria. The objective of this study is to evaluate the ability of *Pseudomonas segetis* strain P6, which was isolated from the *Salicornia europaea* rhizosphere, to act as a potential biocontrol agent given its plant growth-promoting (PGP) and quorum quenching (QQ) activities. Seed biopriming and *in vivo* assays of tomato plants inoculated with strain P6 resulted in an increase in seedling height and weight. We detected QQ activity, involving enzymatic degradation of signal molecules in quorum sensing communication systems, against a broad range of *N*-acylhomoserine lactones (AHLs). HPLC-MRM data and phylogenetic analysis indicated that the QQ enzyme was an acylase. The QQ activity of strain P6 reduced soft rot symptoms caused by *Dickeya solani*, *Pectobacterium atrosepticum* and *P. carotovorum* on potato and carrot. *In vivo* assays showed that the PGP and QQ activities of strain P6 protect tomato plants against *Pseudomonas syringae* pv. tomato, indicating that strain P6 could have biotechnological applications. To our knowledge, this is the first report to show PGP and QQ activities in an indigenous *Pseudomonas* strain from *Salicornia* plants.

## Introduction

Plant bacterial pathogens cause diseases in a wide range of crops worldwide and considerable economic losses in agriculture^1,2^. Antibiotics and chemical pesticides have been used for many decades to combat plant bacterial infections^3,4^. However, stricter legislation has been introduced in recent years regarding the use of chemical-based treatments which have caused serious problems such as reduced productivity due to resistance to treatment, soil salinization and environmental pollution^5^. As a consequence, alternative strategies to combat plant diseases and to promote plant growth are required in order to replace current procedures with more sustainable eco-friendly approaches^6^.

Currently, one of the most promising tools used in the agricultural industry is the use of formulations containing plant growth-promoting bacteria (PGPB), also known as plant growth-promoting rhizobacteria (PGPR). These are beneficial microorganisms that act as biofertilizers and can fight plant pathogens^7,8^. They counteract pathogens through physical displacement, siderophore production, as well as the synthesis of antibiotics, bacteriocins and hydrolytic enzymes which inhibit pathogen growth^8,9^. They also boost plant resistance to infections, which is also called induced systemic resistance (ISR)^10^, through mechanisms such as callose deposition^11^. PGPB, which promote processes such as plant growth and stress tolerance, and can fight phytopathogens, are considered to be an effective, sustainable and environmentally-friendly alternative to be used in agriculture^8,12^. Although several members of the genus *Pseudomonas* have been identified as PGPB, only a few have been isolated from saline environments^13^. Given that soil salinization is an upcoming problem in agriculture due to climate change, salt-tolerant PGPB appear to be a suitable approach to deal with the problem of productivity^14,15^.

Another promising agricultural strategy is the interference of quorum sensing (QS) systems in plant pathogens. QS is an intercellular communication system in which bacterial gene expression, coupled with bacterial cell concentration, is mediated by the diffusion of specific signal molecules such as *N*-acylhomoserine lactones (AHLs)^16^. This system regulates the expression of different phenotpyes, many of which have been shown to contribute to bacterial pathogenesis in a number of economically-important agriculture pathogens^17^. For instance, QS controls numerous phenotypes in *Pectobacterium carotovorum*^18^, *P. atrosepticum*^19,20^, *Pseudomonas syringae*^21^, *Dickeya solani*^22^, *Ralstonia solanacearum*^23^, *Erwinia amylovora*^24^ and *Agrobacterium tumefaciens*^25^. The interruption of QS is therefore an interesting strategy for combating bacterial infections in agriculture^26^. One of the best known QS-interrupting strategies is quorum quenching (QQ) which involves the enzymatic degradation of AHL signal molecules^27^. AHLs can be degraded or modified by different types of enzymes, including lactonases, acylases and oxidorreductases^28^. This strategy has already been reported to reduce the virulence of several plant bacterial pathogens producing promising results^29–31^. Rather than inhibiting growth or killing the pathogen, as occurs with antibiotics, QS attenuates virulence and reduces infection^26,32,33^ without affecting bacterial pathogen growth^29,34^. Given that it does not affect essential bacterial genes^32^ nor leads to the development of resistances, this strategy is though to be more effective at long-term than antibiotics^35,36^, but more studies need to be done to validate such claim. Nevertheless, although some authors have reported the development of resistances to QS interference, they have suggested that these mechanisms would be less prone to spread resistances to other bacteria^37,38^. Interestingly, although many QQ bacteria have been isolated from marine environments^39^, few are indigenous plant isolates and, additionally, their impact on plant growth parameters remains unknown.

The main objective of our study was to analyze the potential of *Pseudomonas segetis* strain P6, a novel strain isolated from *Salicornia* plants, as a biocontrol agent against plant pathogenic bacteria.

## Results

### Characterization of strain P6

In order to characterize strain P6, isolated from the rhizosphere of the halophyte plant *Salicornia europaea*, several morphological, physiological and biochemical studies were conducted. Strain P6 is a motile, Gram-negative, halotolerant rod that is capable of growing in a wide range of salt concentrations [0–7.5% (w/v) NaCl] and according to the Microtox test and the bacterial virulence test using *Artemia salina*, does not show toxicity. It is also able to produce siderophores, as well as enzymes such as lipases, acid and alkaline phosphatases, DNAse, lecithinase and ACC deaminase. With the objective of genetically identifying strain P6, a 1362-bp-fragment of its 16S rRNA gene was sequenced. The analysis showed that strain P6 had 99.93% sequence similarity to *Pseudomonas segetis* FR1439^T^.

### Plant growth-promoting activity

Using biopriming assays, tomato seeds inoculated with *P. segetis* P6 showed significant increases (*P* < 0.01), of 191.8% and 207.0%, in total seedling length and the vigour index, respectively, as compared to the control. The germination rate difference (4.5%) with the negative control was not significant (Table S1). In the case of tomato plants inoculated with strain P6 (Fig. S1), aerial and root dry weight increased sharply (*P* < 0.01) by 19.28% and 21.54%, respectively. Shoot and root length did not increase significantly (they showed modifications of by 7.98% and 13.54%, respectively, *P* ≤ 0.08).

### Characterization of the AHL degradation activity of *P. segetis* strain P6

The QQ activity of strain P6 was evaluated using a wide range of synthetic AHLs. The results indicate that strain P6 totally degraded all the AHLs tested except for 3-O-C6-HSL, which was only partially degraded (Fig. S2).

**Figure 1:**
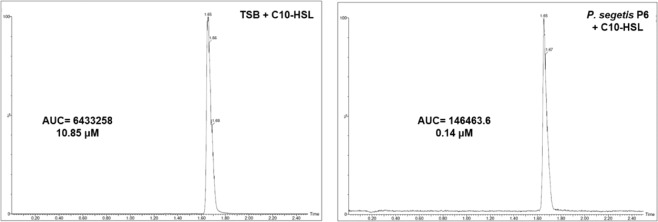
Determination of remaining C10-HSL by HPLC-MRM after 24 h of incubation with *P. segetis* P6. Tryptic soy broth (TSB) was used as a negative control. Initial AHL concentration was 10 µM.

To determine whether the QQ activity of strain P6 was caused by a lactonase-type enzyme, overnight culture supernatants were acidified to pH 2 following incubation with C10-HSL, and the remaining AHLs were detected using HPLC-MRM. AHL extracts from cultures grown under similar conditions, but at pH 7, were also analyzed. The results indicate that AHL degradation in the acidified and neutral reactions was ~99.6% and 98.4%, respectively (Fig. S3). Initial AHL concentration was not restored at pH 2, indicating that the QQ activity of strain P6 could not be caused by a lactonase enzyme. The quantification of remaining C10-HSL by HPLC-MRM (Fig. 1) showed a degradation of 98.7%. Decanoic acid and L-Homoserine lactone, the resulting metabolites of an acylase enzyme degradation were not detected.

In order to identify the enzyme responsible for the QQ activity of P6, we carried out a search for possible genes encoding QQ activity in the genome of *P. segetis* FR1439^T^, which identified the penicillin acylase WP_089360949. Based on its sequence, specific primers were designed and a ~2500 bp fragment was amplified from strain P6, ligated in the pGEM-T vector and transformed into *Escherichia coli* DH5α. The DNA construction was sequenced and the deduced protein sequence confirmed its high homology with other QQ acylase enzymes. To demonstrate the acylase activity, the DNA fragment was also cloned in pGEX-4T-2 vector and expressed in *E. coli* DH5α confirming its AHL-degrading activity on C12-HSL (Fig. S4). A neighbour-joining tree was constructed and phylogenetic analyses showed that the penicillin acylase of *P. segetis* P6 clusters with other penicillin acylases (Fig. S5).

### Interference of bacterial phytopatogen QS systems and impact on associated phenotypes by *P. segetis* strain P6

**Figure 2:**
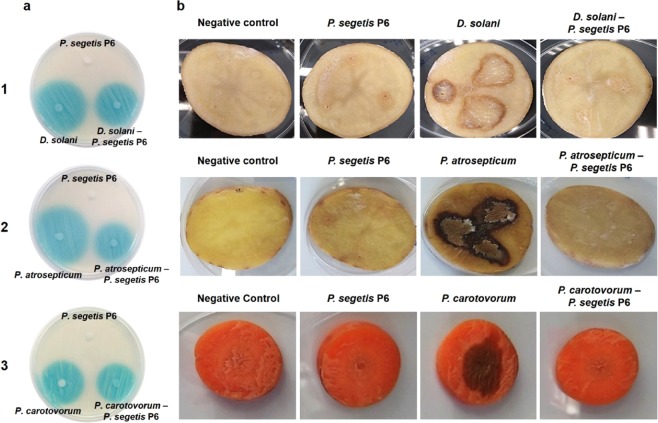
Virulence assay in potato tuber and carrot slices. (**a**) Detection of AHLs in the cultures and co-cultures of *P. segetis* P6 and the pathogens Dickeya solani (1), *Pectobacterium atrosepticum* (2) and *P. carotovorum* subsp. *carotovorum* (3) using the biosensor *Agrobacterium tumefaciens* NTL4. (**b**) Virulence and maceration of cultures and co-cultures of strain P6 and the different pathogens on the surface of potato and carrot slices after 2 days of incubation. Sterile water was used as a negative control.

AHL degradation assays were firstly performed against crude extracts from the bacterial phytopathogens *Dickeya solani*, *Pectobacterium atrosepticum*, *P. carotovorum* subsp. *carotovorum* and *Pseudomonas syringae* pv. tomato. The results obtained indicate that strain P6 fully degraded the AHLs of the phytopathogens (Figs. 2 and S6). Thus, co-cultures were carried out to determine whether *P. segetis* P6 was able to reduce the production of virulence factors controlled by QS in plant bacterial pathogens. An antagonist experiment was first performed to determine whether strain P6 interfered with the growth of the plant pathogens *D. solani*, *P. atrosepticum*, *P. carotovorum* subsp. *carotovorum* and *P. syringae* pv. tomato. The results showed that strain P6 did not have any inhibitory effect on the growth of the pathogens tested (data not shown). Thus, each of the four pathogens was grown in a co-culture with strain P6 in a ratio of 1:100, which was maintained throughout the experiment. The count of each strain was similar to the initial concentration 10^7^–10^8^ CFU mL^−1^. After 24 h of incubation, the AHLs were extracted from the co-cultures. As shown in Fig. 2, the molecules produced by *P. atrosepticum*, *P. carotovorum* subp. *carotovorum* and *D. solani* in the co-cultures activated in less extension the biosensor, while no AHLs were detected in the co-cultures with *P. syringae* pv. tomato DC3000 (Fig. S6).

Under similar conditions, we used the co-cultures and different phenotypic tests to analyze the effect of AHL degradation on the QS-regulated functions of the plant pathogens. Swimming motility and proteolytic activity (caseinase and gelatinase) in *D. solani* were found to be inhibited in the presence of strain P6 (Table S2). Similarly, no caseinase activity was detected in the *P. atrosepticum*-strain P6 co-culture while alkaline phosphatase decreased and swimming motility showed a moderate reduction and an altered pattern (data not shown). Meanwhile, in *P. carotovorum* subsp. *carotovorum* and *P. syringae* pv. tomato, no phenotypes were affected under our test conditions. Nevertheless, as strain P6 produces DNAse and amylase, their interfere by QQ could not be evaluated in the phenotypes of the pathogens (Table S2).

To assess the possible impact of QQ on the virulence and maceration capacity of the phytopathogens tested, we conducted assays of potato and carrot slices. Co-cultures of *D. solani* and *P. atrosepticum* with *P. segetis* P6 were inoculated on the surface of the potato slices. In both cases, the co-culture with strain P6 reduced the capacity of the pathogens to cause soft rot (0% maceration), while a maceration zone of 36.6 ± 2.6% and 65.6 ± 5.9% was observed in the *D. solani* and *P. atrosepticum* monocultures, respectively (Fig. 2). Carrot slices inoculated with the *P. carotovorum* subsp. *carotovorum*-strain P6 co-culture showed no soft rot symptoms (0% maceration) as compared to the 88.4 ± 5.0% maceration produced by *P. carotovorum* subsp. *carotovorum* in the mono-culture. No assay of the strain P6 mono-culture showed soft rot symptoms.

### Plant growth-promoting and QQ activities of strain P6 against *Pseudomonas syringae* pv. tomato

**Figure 3:**
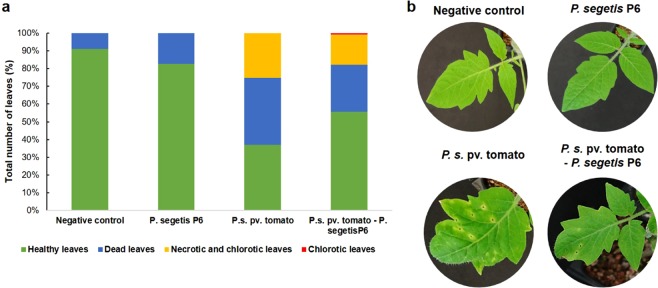
Infection assay in tomato plants treated with cultures and co-cultures of *Pseudomonas syringae* pv. tomato and *P. segetis* P6. (**a**) Total percentage of healthy, dead, necrotic and chlorotic leaves after each treatment. (**b**) Infection symptoms on leaves after treatment.

*In vivo* assays of tomato plants were conducted to evaluate the impact of *P. segetis* strain P6’s plant growth-promoting and QQ activities on the virulence of *P. syringae* pv. tomato. As shown by the number of leaves affected, tomato plants treated with the *P. syringae* pv. tomato-strain P6 co-culture were less damaged than those infected with the pathogen alone (Fig. 3). Tomato plants treated with the co-culture showed a significative increase of 51.0% in healthy leaves as compared to plants inoculated with *P. syringae* pv. tomato. Significative differences were recorded with respect to the number of dead and necrotitc/chlorotic leaves which fell by 29.8% and 32.3%, respectively, as compared to those treated with the pathogen alone. Co-culture-treated plants showed a small number of chlorotic leaves due to a less harmfull form of the pathogen, which was not observed in plants treated with the pathogen alone. Negative control- and strain P6-treated plants showed some dead leaves associated with natural senescence.

Following treatment, the plants were harvested in order to assess the impact of the different treatments on plant growth (Table 1). Total dry weight increased significantly by 84.6% (*P* < 0.01) for plants treated with strain P6 alone. No significant differences in total dry weight between plants treated with *P. syringae* pv. tomato in the presence and absence of strain P6 were observed. However, shoot dry weight increased signficantly by 160.6% (*P* < 0.05) in co-culture-treated plants as compared to those inoculated with *P. syringae* pv. tomato alone. No significant differences in root dry weight were detected in plants inoculated with the pathogen alone or the co-cultures.

**Table 1:** Dry weight of tomato plants after treatment with *Pseudomonas syringae* pv. tomato and *P. segetis* P6.

	Negative control	*P. segetis* P6	*P. syringae* pv. tomato	*P. syringae* pv. tomato - *P. segetis* P6
Root dry weight (mg)	6.38 ± 0.55^a^	13.78 ± 5.53^b^	4.15 ± 0.46^a^	4.76 ± 1.47^a^
Shoot dry weight (mg)	11.82 ± 1.77^a^	19.82 ± 6.49^b^	3.25 ± 0.64^c^	8.47 ± 1.60^a^
Total dry weight (mg)	18.20 ± 1.79^a^	33.60 ± 11.86^b^	7.40 ± 0.69^c^	13.23 ± 3.00^c^

Data are expressed as mean values and standard deviation. Values within a line followed by different letters indicate significant difference (P ≤ 0.05).

**Figure 4:**
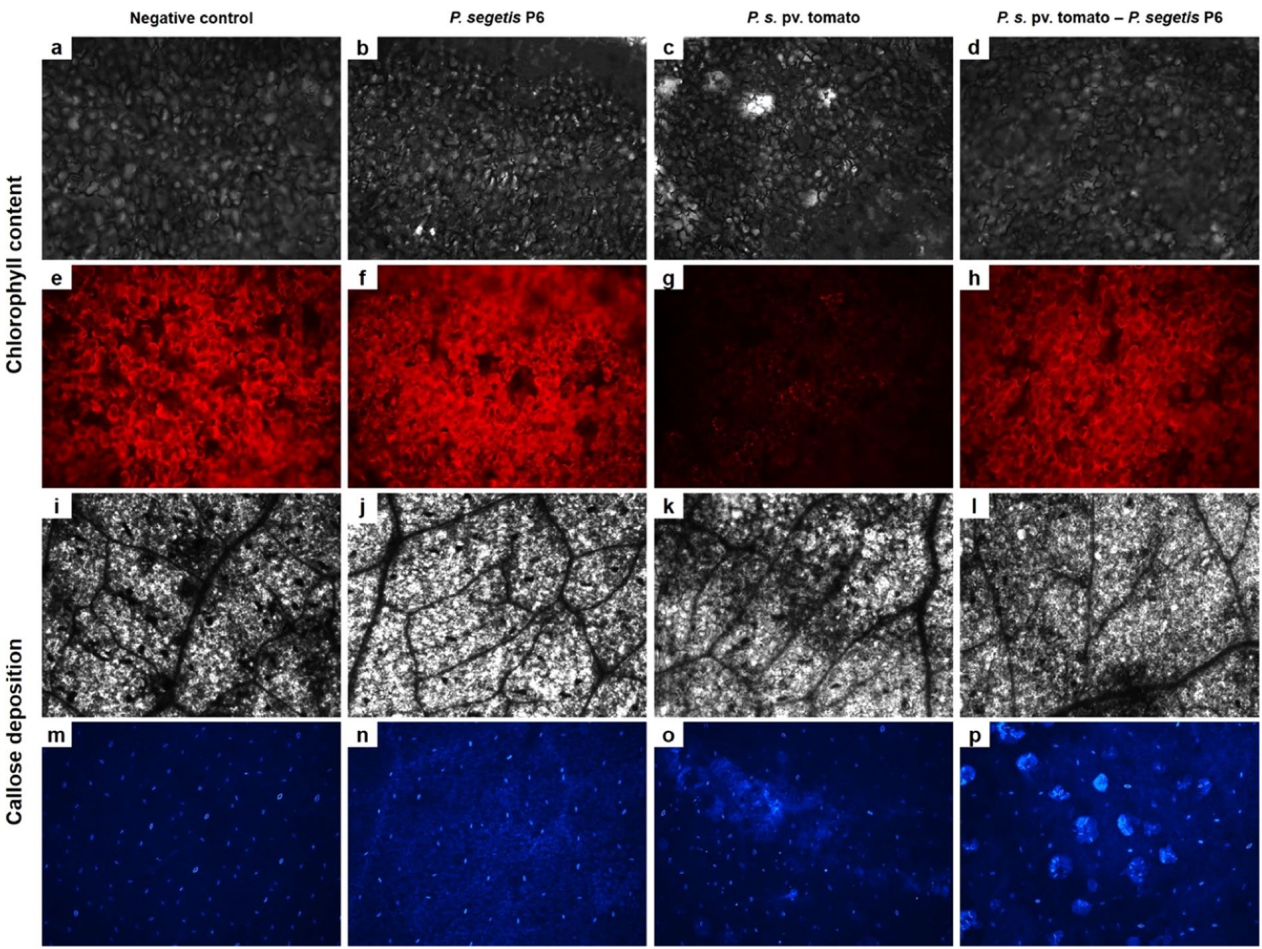
Observation by microscopy of chlorophyll and callose deposits in tomato leaves. Differential interference contrast (DIC) micrographs: (**a–d**) and (**i–l**). Fluorescence micrographs: (**e–h**) and (**m–p**). Each DIC micrograph shows tissue morphology of the corresponding fluorescence micrograph: (**a**–**e**, **b**–**f**, **c**–**g**, **d**–**h**, **i**–**m**, **j**–**n**, **k**–**o** and **l**–**p**). All micrographs from each determination were taken at the same magnification, exposure time, gamma and gain settings. Fluorescence micrographs were digitally colored.

In order to evaluate the impact of *P. syringae* pv. tomato on photosynthetic plant tissue, chlorophill *a* and *b* and total chlorophill content were determined using Arnon and Lichtenthaler’s equations. The results obtained for plants treated with strain P6 showed higher levels of total chlorophyll with respect to the other treatments (Table 2). Total chlorophyll levels in the co-culture-treated plants were very similar to those of negative controls and slightly higher than those in the plants infected with the pathogen alone. However, the differences observed with the aid of spectrophotometry were not statistically significant, which is probably due to the low resolution of this method of determination. Fluorescence microscopy (Fig. 4) revealed differences between treatments with regard to chlorophyll content, as well as chloroplast abundance and integrity. Leaves treated with *P. segetis* P6 showed larger amounts of chlorophyll as compared to the other treatments. Differences were observed between plants treated with the *P. syringae* pv. tomato monoculture and those treated with the strain P6 co-culture. Lower chlorophyll and chloroplast concentrations surrounding cell walls were observerd in plants treated with the pathogen alone. Using DIC microscopy, *P. syringae* pv. tomato-treated leaves showed an altered tissue structure, with the appearance of some clear spots which were absent in the other leaves (Fig. 4).

**Table 2:** Chlorophyll content of fresh shoots after treatment with *Pseudomonas syringae* pv. tomato and *Pseudomonas segetis* P6.

	Parameter	Negative control	*P. segetis* P6	*P. syringae* pv. tomato	*P. syringae* pv. tomato -*P. segetis* P6
Arnon formula	Chlorophyll *a* (mg g^−1^)	0.52 ± 0.14	0.63 ± 0.13	0.41 ± 0.08	0.57 ± 0.12
	Chlorophyll *b* (mg g^−1^)	0.20 ± 0.05	0.27 ± 0.05	0.17 ± 0.03	0.23 ± 0.12
	Total chlorophyll (mg g^−1^)	0.72 ± 0.19	0.91 ± 0.19	0.58 ± 0.12	0.80 ± 0.42
Lichtenthaler formula	Chlorophyll *a* (mg g^−1^)	0.50 ± 0.13	0.60 ± 0.13	0.39 ± 0.08	0.55 ± 0.29
	Chlorophyll *b* (mg g^−1^)	0.14 ± 0.03	0.19 ± 0.04	0.12 ± 0.02	0.16 ± 0.08
	Total chlorophyll (mg g^−1^)	0.64 ± 0.16	0.79 ± 0.16	0.51 ± 0.10	0.70 ± 0.37

Measurements were carried out using the Arnon and Lichtenthaler methods. Data are expressed as mean values and standard deviation.

Callose deposition was analysed in fresh shoots to evaluate the stimulation of defense mechanisms. The results obtained (Fig. 4) revealed callose deposition in leaves treated with strain P6, while no deposition was observed in negative control leaves. Leaves treated with the pathogen-strain P6 co-culture also showed callose deposits, with strong deposition surrounding the stomatal guard cells. In plants treated with the *P. syringae* pv. tomato monoculture, callose deposition was detected in the form of a diffuse veil near the spot-affected area.

## Discussion

Novel strategies to boost plant growth and combat plant diseases in agriculture are currently being investigated with the aim of replacing current procedures with more sustainable approaches^6^. Many PGPB, such as *Pseudomonas* spp., act as biological control agents against plant diseases^40^, produce compounds including hormones, antibiotics, polysaccharides and siderophores, induce ISR in plants and increase their abilitiy to defend against pathogens^41–43^.

Likewise, the enzymatic degradation of AHL signal molecules in plant pathogenic bacteria appears to be an interesting alternative strategy for combating bacterial infections^26^. As interference with AHL activity reduces QS-regulated phenotypes, including certain virulence factors, without affecting pathogen growth^29,34^, resistance development is less probable given that essential bacterial genes are unaffected^38^.

Although many PGPB and QQ bacteria have been described^8,44^, no studies have been carried out on a strain that combines both plant growth promotion and AHL degradation or, to our knowledge, on the silencing of bacterial phytopathogen virulence by a PGP bacterium to degrade QS-signalling molecules. In this study, we evaluate the use of strain P6’s PGP and QQ activities as a beneficial strategy to promote plant growth and to control bacterial infections. Strain P6, which had been isolated from the *Salicornia europaea* rhizosphere, belongs to the species *Pseudomonas segetis*^45^. To our knowledge, this is the first time that this species has been isolated from a saline environment.

Although we found no information regarding *P. segetis*^45^, this species belongs to a genus commonly found among PGPB. Capable of growing in a wide range of NaCl concentrations, with optimal growth occurring at 1% (w/v) NaCl, *P. segetis* is classified as a halotolerant bacterium. This boosts its biotechnological potential in agriculture, which is increasingly affected by climate change, for which salt-tolerant PGPB represent a promising solution^15,41^. Several PGPB genera, such as *Azospirillum, Arthrobacter, Bacillus, Burkholderia, Enterobacter* and *Pseudomonas*, are already being used to improve salt tolerance in agriculture crops^46^.

We evaluated the plant-growth promotion properties of *P. segetis* P6 using biopriming techniques and *in vivo* experiments with tomato plants under sterile conditions. The results indicated that strain P6 substantially affects all the parameters studied, with an increase in plant length and vigor index observed in seeds and an increase of weight in plants treated by strain P6 with respect to negative controls. Given the sterile conditions under which the experiments were carried out, the plant growth promotion observed was caused by strain P6 alone rather than in conjunction with other soil bacteria. As reported for other *Pseudomonas* spp. bacteria, this is probably due to strain P6’s PGP properties such as acid/alkaline phosphatase and siderophore production and nitrogen fixation^47,48^. The PGP ability of several *Pseudomonas* species has been evaluated by other authors. For instance, tomato seed biopriming with *P. aeruginosa* and with several fluoresecent *Pseudomonas* spp. increased root lenght by 100% and vigor index by 138–177%^49,50^. On the other hand, addition of *P. geniculata* in tomato plants enhanced aerial and root weight by 7 and 9%, respectively^51^, while *P. fluorescens* increased aerial dry weight by 4.7%^52^. These results contrast with the increase in plant length, vigor index and aerial weight by ~191, 207% and ~19% produced by strain P6, respectively. Regarding the species *P. segetis*, there are no studies focusing on plant growth promotion to which compare our data.

The QQ activity of *P. segetis* P6, which was evaluated against a wide range of synthetic AHLs with different chemical substitutions, resulted in efficient short-, medium- and long-chain AHL degradation. Despite a more frequent degradion of long-chain AHLs among AHL-degrading bacteria^53–55^, strain P6 totally or partially degraded all the molecules assayed.

To identify the type of QQ in *P. segetis* P6, the samples were acidified and incubated in the presence of AHLs^56^. Acidification enabled the AHL lactone ring to restructure itself if previously degraded by lactonase. No significant recovery in the AHLs tested was observed using HPLC-MRM analysis, suggesting that the degradation activity was not caused by lactonase. This result was confirmed by the quantification of remaining C10-HSL by HPLC-MRM which showed a total degradation of this AHL. Our data suggest that the QQ enzyme can be an acylase although the corresponding metabolites decanoic acid and *L*-Homoserine lactone metabolites were not detected in HPLC-MRM analysis, probably due to the incorporation of them in the metabolic activity of *P. segetis* P6. As the *P. segetis* FR1439^T^ genome search showed a putative QQ enzyme annotated as a penicillin acylase, a similar gene was amplified and sequenced in strain P6. A phylogenetic analysis found that its predicted amino acid sequence co-clustered with other QQ acylase enzymes, which reinforces the hypothesis of the presence of an acylase enzyme in strain P6. This gene was cloned into pGEX-4T-2 and expressed in *E. coli* DH5α demonstrating the AHL degradation activity of P6 strain. Although previous studies have reported that penicillin acylases are capable of degrading AHLs in other bacteria^57,58^, this activity had not previously been identified in the species *Pseudomonas segetis*. The actual physiological significance of AHL-degrading enzymes remains largely unclear^59^. Several authors have suggested that AHL degradation could be related with a self-regulation of intercellular systems^25^. Using different AHL biosensor strains we found that strain P6 does not produce any AHLs, nor could we identify any gene coding for a QS signal synthase (*luxI*) in the genome of the type strain *P. segetis* FR1439^T^. This shows that the AHL-degrading capacity of P6 is not associated to self-regulating intercellular systems. However, we found an orphan QS signal receptor/regulator (*luxR*). It is emerging that the *luxR* orphans could allow bacteria to respond to endogenous and exogenous signals produced by their neighbors^60^.

We also tested *P. segetis* P6’s capacity to degrade AHL-containing crude extracts from major plant bacterial pathogens such as *Dickeya solani*, *Pectobacterium atrosepticum*, *P. carotovorum* subsp. *carotovorum* and *P. syringae* pv. tomato. This capacity was confirmed by experiments with plant bacterial pathogen-strain P6 co-cultures. Given that AHLs regulate the expression of some virulence factors in many plant bacterial pathogens^17^, the results obtained show that strain P6 could be a potential candidate for controlling bacterial infections in agriculture through enzymatic inhibition of QS systems.

The impact of AHL degradation on the production of virulence factors could not be proved for some plant pathogens in the co-culture experiments, as *P. segetis* P6 itself produces many hydrolytic enzymes. However, we did observe some effects on *D. solani*, in which caseinase, gelatinase and motility were totally inhibited in the co-culture with strain P6, while lipase (hydrolysis of Tween 80) was reduced by ~70%, though not inhibited. Similar results were found for *P. atrosepticum* when co-cultured with strain P6, resulting in an inhibition of caseinase and a substantial reduction in swimming motility. Moreover, experiments on potatoes and carrots demonstrated that the addition of strain P6 significantly reduced soft-rot maceration caused by *D. solani*, *P. atrosepticum* and *P. carotovorum* subsp. *carotovorum*. This reduction in pathogen virulence was due to the QQ activity of strain P6 and not to any inhibitory effect on pathogen growth, as the concentration of each bacterium was maintained throughout the experiments. Several studies have also demonstrated that AHL-degrading bacteria prevent and reduce pathogen QS-dependant plant infection^30,31,54,61^. The heterologous expression of QQ enzymes in bacterial pathogens, such as *P. carotovorum* subsp. *carotovorum*^22,62,63^ and *D. chrysanthemi*^64^, also reduces AHL accumulation and virulence.

*P. segetis* P6’s combination of PGP and QQ activities was evaluated by performing indoor greenhouse experiments on tomato plants infected by *P. syringae* pv. tomato strain DC3000. A significant reduction was observed in the number of dead leaves and necrotic/chlorotic symptoms in the plants treated with the *P. syringae* pv. tomato DC3000-strain P6 co-culture. Additionally, the higher chlorofill content detected by fluorescence microscopy demonstrates that strain P6 protects against the bacterial speck caused by *P. syringae* pv. tomato in tomato plants. Plants treated with the co-culture or with strain P6 alone showed an increase in shoot dry weight as compared to plants inoculated with the pathogen alone. Given that *P. syringae* mainly affects shoot photosynthetic tissue (leaves, stems, etc) but not roots^65^, this increase reinforces the protective effect of strain P6 against infection by the pathogen.

Callose deposition was also evaluated in order to determine the biocontrol potential of *P. segetis* P6. Callose has been proven to play a defensive role in plants by reinforcing cell walls and by hampering or controlling pathogen infections^66^. Some PGPB of the genera *Bacillus* and *Pseudomonas* have been reported to induce callose deposition via ISR^67,68^, which activates basal defense reponses prior to pathogen infection. As pathogen-associated molecular patterns (PAMPs), such as flagellin22 (flg22) and coronatine (COR), produced by *P. syringae* pv. tomato also induce callose deposits^69^, callose deposition caused by the treatment of tomato plants with strain P6 could be due to the induction of basal defenses. This is corroborated by strong callose deposition in cells surrounding stomatal guard cells in co-culture-treated leaves, as *P. syringae* pv. tomato infection first entered via the stomata and then disseminated through the leaf tissue. These callose deposits surrounding stomata act as a first line of defense against *P. syringae* pv. tomato infection and appear to be induced by strain P6 in order to reinforce the cell wall. These findings, together with a reduced incidence of disease in pathogen-strain P6 co-culture-treated plants suggest that this strain could be used as a safe environmentally-friendly alternative to pesticides commonly used in agriculture.

In previous studies, PGPB species, such as *Lysobacter enzymogenes*^70^ and *Pseudomonas putida*^71^, were transformed by an AHL-degrading gene, which led to a substantial reduction in *P. carotovorum* soft rot symptoms in Chinese cabbage and potatoes. However, to our knowledge, no naturally occurring AHL-degrading PGPB were identified prior to our study.

In summary, we used both *in vitro* and *in vivo* experiments to demonstrate that *Pseudomonas segetis* strain P6 is a plant growth-promoting quorum-quenching bacterium. The results obtained suggest that strain P6 has great potential as a biocontrol agent in the agriculture sector.

## Experimental procedures

### Bacterial strains, media, compounds and culture conditions

Strain P6 was isolated from the rhizosphere of the salt-tolerant plant *Salicornia europaea*. Strain P6 and the phytopathogen strains *Dickeya solani* LMG 25993^T^*, Pectobacterium carotovorum* subsp. *carotovorum* CECT 225^T^*, P. atrosepticum* CECT 314^T^ and *Pseudomonas syringae* pv. tomato DC3000 were grown in tryptic soy broth (TSB) medium. The biosensor strain *Chromobacterium violaceum* CV026^72^ was used to detect C4- to C8-HSL, while *C. violaceum* VIR07^73^ was used to detect C10- and 3-OH-C10-HSL. We used *Agrobacterium tumefaciens* NTL4 (pZLR4) to detect C8- to C12-HSL. Biosensors CV026 and VIR07 were grown in Luria-Bertani (LB) medium, while NTL4 was grown in *Agrobacterium* broth (AB) medium^74^. When necessary, the antibiotics kanamycin (Km) and gentamicin (Gm) were used in final concentrations of 50 μg mL^−1^. Unless otherwise stated, all strains were grown at 28 °C and at 120 rpm in a rotary shaker.

The synthetic AHLs (Sigma-Aldrich, Saint Louis, USA) used were: C4-HSL (*N*-butyryl-DL-homoserine lactone), C6-HSL (*N*-hexanoyl-DL-homoserine lactone), 3-O-C6-HSL (*N*-3-oxo-hexanoyl-DL-homoserine lactone), C8-HSL (*N*-octanoyl- DL -homoserine lactone), 3-O-C8-HSL (*N*-3-oxo-octanoyl-DL-homoserine lactone), C10-HSL (*N*-decanoyl-DL-homoserine lactone), 3-OH-C10-HSL (*N*-3-hydroxydecanoyl-DL-homoserine lactone), C12-HSL (*N*-dodecanoyl-DL-homoserine lactone) and 3-O-C12-HSL (*N*-3-oxo-dodecanoyl-DL-homoserine lactone).

### Characterization of strain P6

Optimum salinity growth conditions were determined in TSB medium supplemented with different concentrations of NaCl [0.5, 1, 3, 5, 7.5 and 10% (w/v)]. The following phenotypic characteristics were evaluated: acid and alkaline phosphatase production^75,76^; hydrolysis of starch, casein, DNA, Tween 20, Tween 80 and gelatin^77,78^; siderophore production^79^ and swimming motility^80^. Virulence was evaluated using the *Artemia salina* infection model^81^ and ecotoxicity was assessed by a *Aliivibrio fischeri* bioluminescence inhibition assay^82^.

Genomic DNA of strain P6 was isolated using the XDNA purification kit (Xtrem Biotech, Granada, Spain). The 16S rRNA gene was amplified using primers 16F27 and 16R1488^83^ and the PCR product was sequenced and compared to reference 16S rRNA gene sequences available in the NCBI database using the BLASTN search tool^84^, while pairwise 16S rRNA gene sequence similarity was calculated using the EzBioCloud server^85^.

### Plant growth promotion assays

PGP capacity of strain P6 was tested by biopriming tomato (*Solanum lycopersicum*) seeds and was also assessed in plant seedlings cultured in pots under sterile conditions^86,87^. In both cases, tomato seeds were surface-sterilized according to the protocol described by Molan *et al*.^88^. Biopriming assay was performed as previously described^89^. Vigour index, germination rate and length of germinated plants were evaluated.

For plant growth promotion assays, 50 sterilized seeds were sown in each 20 × 20 × 20 cm pot containing sterile vermiculite. When seedlings reached 5 cm, each pot was irrigated with 10 mL of 10^9^ CFU mL^−1^ of strain P6-washed cells every seven days. SDW was used for the negative controls. Pots were kept in an indoor greenhouse during a long-day photoperiod (16:8 h light:dark) at 25 °C for 4 weeks. Then, root and shoot length and dry weight were determined.

### Quorum-quenching activity against synthetic AHLs and crude AHL extracts from plant bacterial pathogens

Strain P6’s QQ activity was analysed using a well diffusion agar-plate assay^90,91^. Briefly, overnight grown strain P6 cultures were supplemented with 10 µM of each AHL and then incubated at 28 °C for 24 h. Cell-free TSB medium supplemented with AHLs was incubated as a negative control. The remaining AHLs were detected on LB agar plates overlaid with CV026 or VIR07, or on AB agar plates supplemented with 80 µg mL^−1^ of 5-bromo-4-chloro-3-indolyl-ß-D-galactopyranoside (Xgal) overlaid with NTL4 to check for the appearance of a purple or blue color around each well.

To evaluate QQ activity against the phytopathogen-produced AHLs, crude AHL extracts from each pathogen strain were obtained as described elsewhere^92,93^. Briefly, AHLs were extracted twice with equal volumes of dichloromethane, dried, and finally suspended in 20 µL of 70% (v/v) methanol. Then, each crude AHL extract was added to an overnight culture of strain P6 and incubated at 28 °C for 24 h. The remaining AHLs in the whole culture were extracted as explained above, spotted on sterile 5-mm-diameter paper disks placed on AB agar plates and detected with NTL4 as explained above.

### Characterization of the AHL-degrading enzyme

An assay based on lactone ring closure following acidification was performed to elucidate the QQ activity type of strain P6^56,94^. Briefly, C10-HSL (10 µM) was added to an overnight culture of strain P6 and incubated for 24 h at 28 °C. As a negative control, cell-free TSB medium was supplemented with the same AHL concentration. Then, an aliquot of supernatant was acidified to pH 2 using HCL 1M and incubated for 24 h. The remaining AHLs were extracted and qualitatively and quantitatively measured using the well diffusion-agar plate method and high-performance liquid chromatography-multiple reaction monitoring (HPLC-MRM), respectively^53^.

The genome of *P. segetis* FR1439^T^ (NZ_FZOG01000001) was used for an *in silico* search for reference genes encoding for QQ activity. A gene coding for a predicted acylase was selected and used to design the following specific primers: forward 5′-ATGCAATCGCGTGTGTTTCG-3′ and reverse 5′-TTATTTGCCGGGCGTGAGC-3′. The gene was amplified by PCR in strain P6, purified and cloned into the pGEM-T cloning vector. Neighbour-joining tree was performed for the phylogenic reconstruction of the acylase sequence. The putative acylase gene was also amplified with primers psacBamHI-F 5′-GGATCCATGCAATCGCGTG-3′ and psacECoRI-R 5′-GAATTCTTATTTGCCGGGC-3′ (*Bam*HI and *Eco*RI sites are underlined) and ligated in the expression vector pGEX-4T-2, previously digested with the appropriate enzymes. This construction was cloned in *E. coli* DH5α and the activity of the clones were tested.

### Antagonist assay

Antagonistic activity of strain P6 against the phytopathogens used in this study was evaluated using the well diffusion method^95^.

### Interference with the QS system of phytopathogens by co-culture assays

Co-culture assays of phytopathogens and strain P6 were performed according to the methodology described by Torres *et al*.^96^. Briefly, pathogen (10^7^ CFU mL^−1^)-strain P6 (10^9^ CFU mL^−1^) co-cultures were conducted in a 1:100 ratio in TSB medium and incubated for 24 h at 28 °C. A similar concentration of each pathogen was added to cell-free TSB as negative control. The remaining AHLs from each co-culture were quantified according to the well diffusion-agar plate method using NTL4. The abundance of each bacterium was determined in the co-culture by serial dilutions and plate counts using TSA and MY medium^97^ modified by a balanced mixture of 5% (w/v) sea salt solution^98^.

### Virulence assays in potato and carrot slices

The ability of strain P6 to interfere with soft rot caused by *D. solani* LMG 25993^T^ and *P. atrosepticum* CECT 314^T^ was assessed on potato slices, and on carrot slices for *P. carotovorum* subsp. *carotovorum* CECT 225^T^ ^61,63^. Briefly, surface-sterilized and sliced potato tuber (*Solanum tuberosum*) and carrots (*Daucus carota*) were inoculated with P6-phytopathogen co-cultures and controls (the bacterium mono-cultures and the SDW). Nine replicates of each treatment were performed, and the experiment was repeated three times. After 48 h of incubation at 28 °C, maceration zones were visually detected, and the spatial extent of the damage was calculated using ImageJ software^99^. Plate counts in co-cultures were performed to assess the concentration of each phytopathogen and strain P6.

### *In vivo* tomato plant virulence test

The effect of AHL degradation caused by strain P6 on *P. syringae* pv. *tomato* DC3000 virulence was tested in tomato plants according to the technique described by Yan *et al*.^87^. Briefly, tomato seeds were surface-sterilized and sown in pots with vermiculite as described above. Four treatments were tested: sterile distilled water (negative control), *P. syringae* (positive control), strain P6 and the strain P6-*P. syringae* co-culture. Three pots, each containing 50 tomato seeds, were used per treatment. Plants treated with strain P6 (alone or in co-culture) were inoculated with 10 mL of washed cells of strain P6 (10^9^ CFU mL^−1^). This was done every seven days for three weeks to enable strain P6 to establish in soil and to interact with the seedlings. The other pots were inoculated only with 10 mL of SDW. Pots were kept in an indoor greenhouse during a long-day photoperiod (16:8 h light:dark) at 25 °C for four weeks. After the third week, the pots were exposed to 100% humidity for 16 h to induce stomatal opening. Then, the pots to be infected with the pathogen (alone or in co-culture) were sprayed with 5 mL of *P. syringae* pv. tomato-washed cells (10^9^ CFU mL^1^). Relative humidity was maintained at 100% for 24 h to facilitate pathogen infection.

After a week post-inoculation, leaf infection symptoms were recorded^100^ from a total of 400 leaves. The shoot and root length and total dry weight of 20 plants per treatment were determined. Chlorophyll content was determined in 100 mg of fresh shoots by acetone extraction and absorbance reading^101–103^ and with a fluorescence microscope at wavelength 365 nm^104^. Callose deposition was studied in fresh shoots using fluorescence microscopy after ethanol decoloration and methylene blue coloration^105^. Differential interference contrast (DIC) microscopy was used to visualize tissue morphology. Assays were conducted in triplicate.

### Statistical analysis

Statistical analyses were carried out using Statistical Package for the Social Sciences (SPSS) software. Data normalization was checked by the Shapiro-Wilk test. Finally, we used ANOVA and post-hoc Tukey analyses to assess the effects of each treatment.

## Supplementary information

Supplementary information.
