# Supplementary material for: Plant growth-promoting activity and quorum quenching-mediated biocontrol of bacterial phytopathogens by Pseudomonas segetis strain P6
Source: Sci Rep. 2020 Mar 5;10:4121. doi: 10.1038/s41598-020-61084-1 (PMC7058018; doi:10.1038/s41598-020-61084-1)
Supplement: Supplementary file 1 — Supplementary information. [file 41598_2020_61084_MOESM1_ESM.docx]

**Plant growth-promoting activity and quorum quenching-mediated biocontrol of bacterial phytopathogens by *Pseudomonas segetis* strain P6**

Miguel Rodríguez^1^, Marta Torres^1,2,3^, Lydia Blanco^1^, Victoria Béjar^1,2^, Inmaculada Sampedro^1^*, Inmaculada Llamas^1,2^*

^1^Department of Microbiology, Faculty of Pharmacy, University of Granada, Granada, Spain

^2^Institute of Biotechnology, Biomedical Research Center (CIBM), University of Granada, Granada, Spain

^3^Institute for Integrative Biology of the Cell (I2BC), CEA/CNRS/University Paris-Sud, University Paris-Saclay, 91198 Gif-sur-Yvette, France

*Author for correspondence: Inmaculada Llamas (I.L) and Inmaculada Sampedro (I.S) Department of Microbiology, Faculty of Pharmacy, Campus de la Cartuja s/n, Granada, Spain. 958249935. [illamas@ugr.es](mailto:illamas@ugr.es); [isampedro@ugr.es](mailto:isampedro@ugr.es)


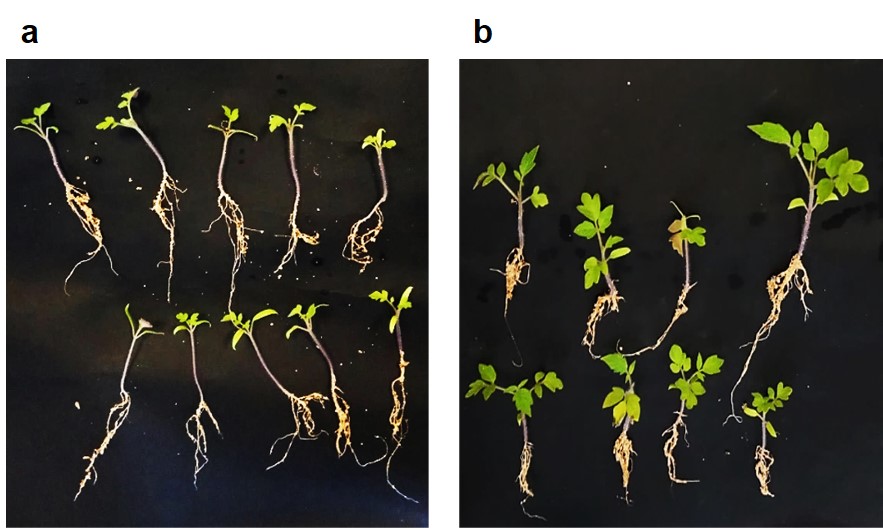


**Figure S1.** Tomato seedlings from plant-growth promoting assay after 4 weeks. (a) Tomato plants treated with sterile distilled water, (b) tomato plants inoculated with strain P6.


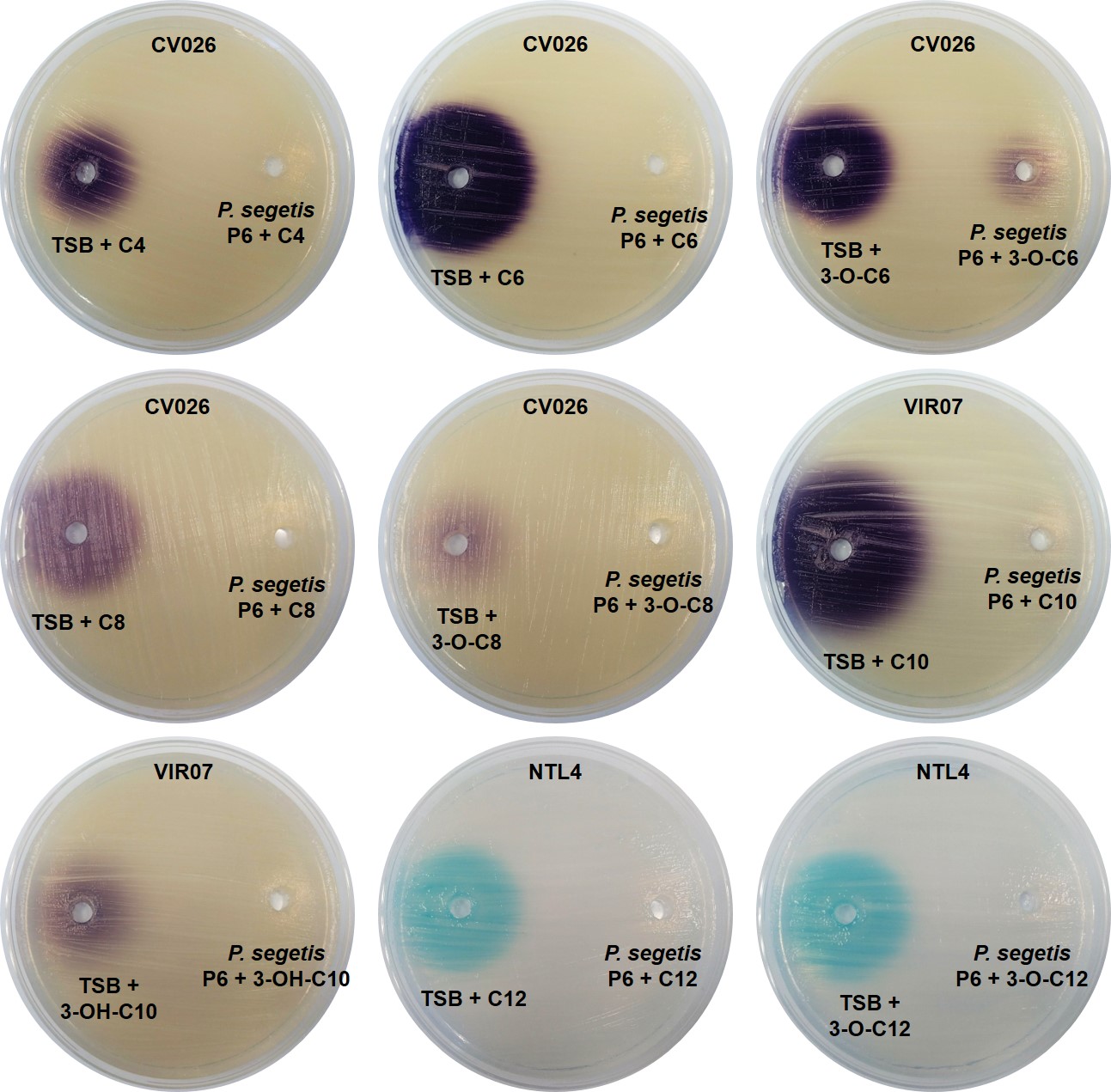


**Figure S2.** Synthetic AHL degradation assay based on diffusion agar-plate method. Chromobacterium violaceum CV026, C. violaceum VIR07 and Agrobacterium tumefaciens NTL4 (pZLR4) were used as biosensor strains. Left wells of each plate constituted controls and contained trypticase soy broth (TSB) medium supplemented with 10µM of each AHL, while right wells contained the supernatant of 24h incubation culture of strain P6 in TSB medium supplemented with the same concentration of each AHL.

**
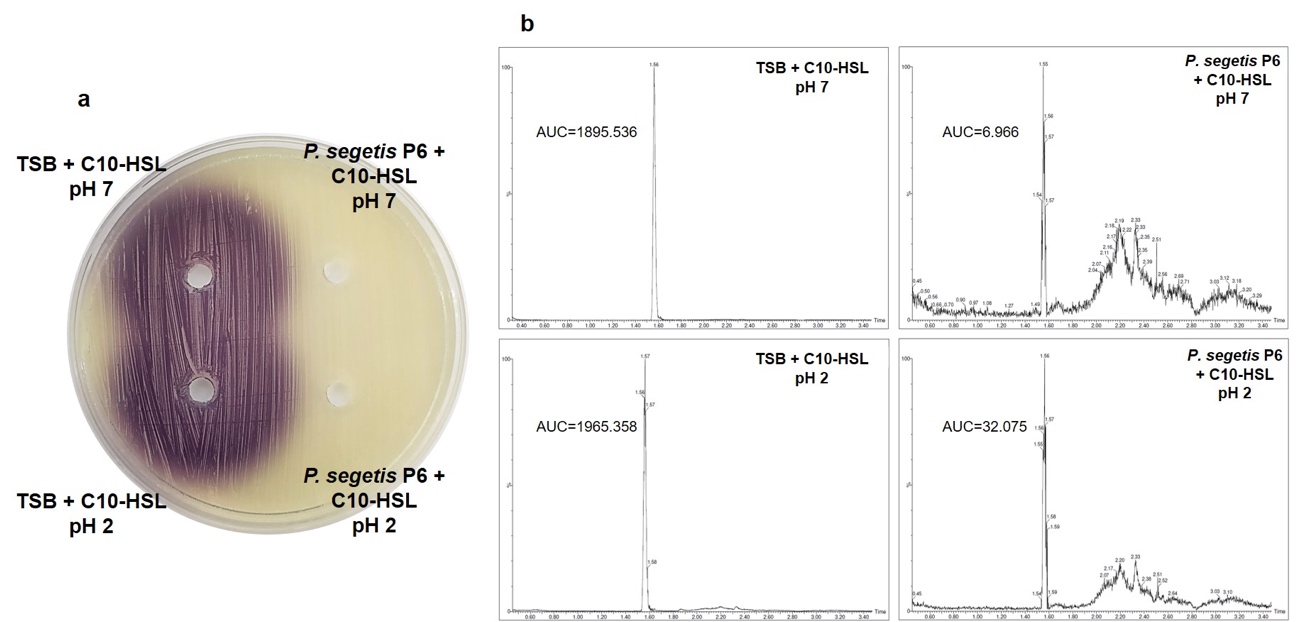
**

**Figure S3. Characterization of Pseudomonas segetis P6 quorum quenching activity under neutral and acidic conditions.** **a**. Diffusion-agar plate assay to detect C10-HSL using biosensor Chromobacterium violaceum VIR07. **b**. HPLC/MS measurement of remaining C10-HSL after 24 h of incubation with P. segetis P6. Tryptic soy broth (TSB) was used as a negative control. Initial AHL concentration was 10 µM.


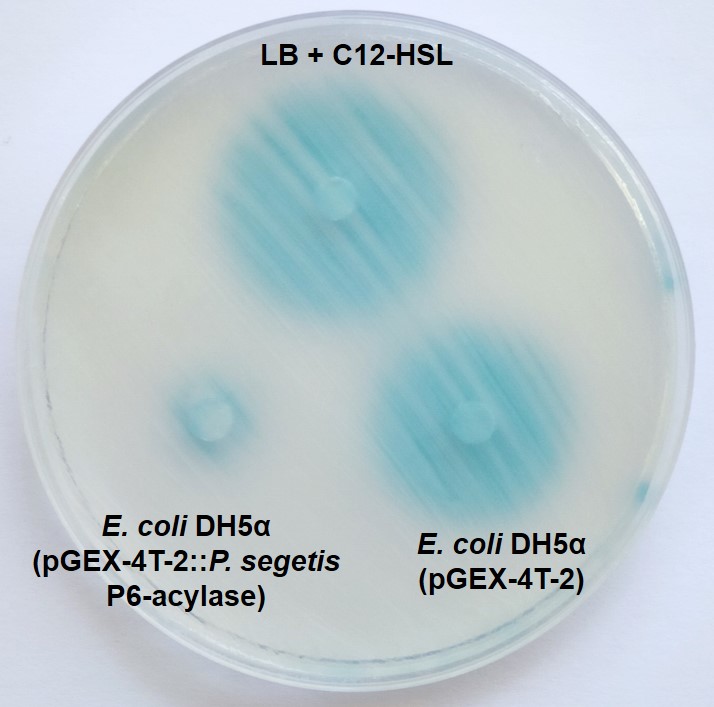


**Figure S4.** C12-HSL degradation assay of P. segetis P6 acylase cloned into pGEX-4T-2 after heterologous expression in E. coli DH5α. LB medium supplemented with C12-HSL was used as control and Agrobacterium tumefaciens NTL4 (pZLR4) was used as biosensor strain.


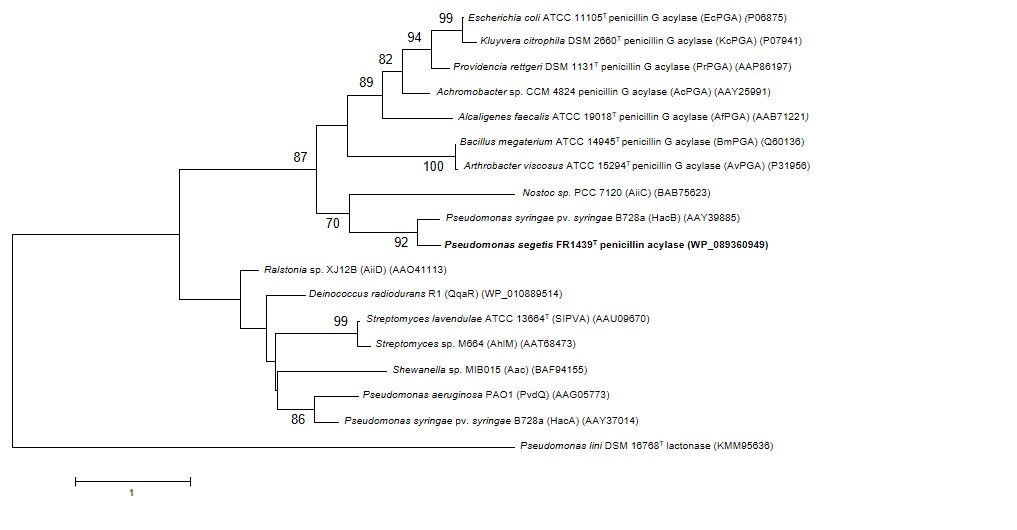


**Figure S5**. Phylogenetic reconstruction of Pseudomonas sp. P6 acylase sequence and the main type of acylase enzymes obtained by neighbour-joining algorithm. Genbank accesion number of each sequence is shown in parenthesis. Bootstrap values are expressed as percentages of 1,000 replications, and those over 70% are shown at branch points. Bar indicates 1 substitution per amino acid position. The Pseudomonas lini DSM 167768^T^ lactonase sequence was used as outgroup.


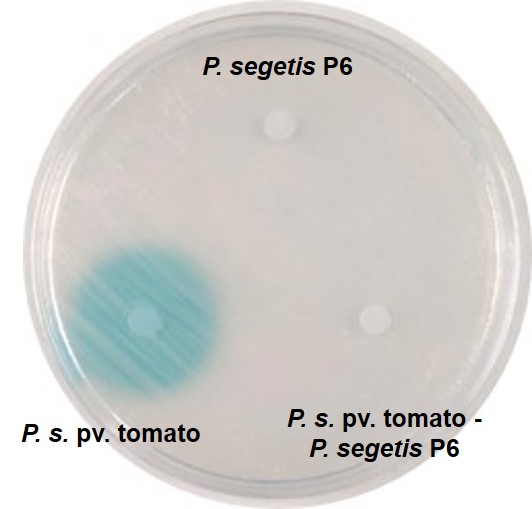


**Figure S6.** *Pseudomonas syringae* pv. tomato DC3000 strain AHL degradation assay in coculture with *P. segetis* P6. *Agrobacterium tumefaciens* NTL4 (pZLR4) was used as biosensor strain.

**Table S1.** Effects on tomato seeds and seedlings after treated with strain P6 in biopriming and plant growth-promoting assays. Values are expressed as mean ± SD and asterisks means significantly differences between treatments with p<0.05 after ANOVA and Tukey tests. All tests were two-tailed and p values were: <0.0001; 0.1154; 0.0067; 0.0557; 0.0851; 0.0101 and 0.0038, respectively.

|  | **Parameter** | **Control** | **P6 treatment** |
| --- | --- | --- | --- |
| Biopriming | Total length (cm) | 1.59 ± 0.33 | 4.64 ± 0.84* |
|  | Total length increase (%) | - | 191.82 |
|  | Germination rate (%) | 92.30 ± 1.00 | 96.46 ± 3.45 |
|  | Germination rate increase (%) | - | 4.51 |
|  | Vigour index | 146.37 ± 30.98 | 449.32 ± 96.71* |
|  | Vigour index increase (%) | - | 206.98 |
| Inoculation | Root length (cm) | 6.35 ± 1.63 | 7.21 ± 0.59 |
|  | Root length increase (%) | - | 13,54 |
|  | Shoot length (cm) | 4.76 ± 0.88 | 5.14 ± 0.42 |
|  | Shoot length increase (%) | - | 7.98 |
|  | Root dry weight (mg) | 3.76 ± 0.9 | 4.57 ± 0.79* |
|  | Root dry weight increase (%) | - | 21,54 |
|  | Shoot dry weight (mg) | 6.38 ± 1.24 | 7.61 ± 1.03* |
|  | Shoot dry weight increase (%) | - | 19,28 |

**Table S2.** Phytopathogen quorum sensing disruption by strain P6 under coculture conditions. V, variable result; -, negative result; +, weak activity; ++, moderate activity; +++, strong activity.

|  | **Acid phosphatase** | **Alkaline phosphatase** | **Amylase** | **Caseinase** | **DNAse** | **Gelatinase** | **Siderophores** | **Swimming motility** | **Tween^®^ 20** | **Tween^®^ 80** |
| --- | --- | --- | --- | --- | --- | --- | --- | --- | --- | --- |
| *P. segetis* P6 | + | + | + | - | + | - | + | - | + | + |
| *D. solani* | - | ++ | + | + | - | + | + | +++ | + | +++ |
| *D. solani* - *P. segetis* P6 | + | + | + | - | + | - | + | - | + | + |
| *P. atrosepticum* | - | +++ | + | + | - | - | + | +++ | + | +++ |
| *P. atrosepticum* - *P. segetis* P6 | + | + | + | - | + | - | + | ++ | + | + |
| *P. carotovorum* | + | - | + | + | + | + | + | - | + | - |
| *P. carotovorum* - *P. segetis* P6 | + | + | + | + | + | + | + | - | + | + |
| *P.s.* pv *tomato* | + | - | - | - | - | - | - | - | + | + |
| *P.s.* pv tomato - *P. segetis* P6 | + | + | + | - | + | - | + | - | + | + |
